# Interfacing a titrator to a microcomputer for incremental or continuous modes of operation

**DOI:** 10.1155/S1463924680000582

**Published:** 1980

**Authors:** M. Dancziger, H. V. Malmstadt

**Affiliations:** School of Chemical Sciences University of Illinois Urbana Illinois 61801 USA


### Conclusion

It has been demonstrated that the electrochemical detection of iodine in the presence of excess iodide can be reliable and sensitive, when the appropriate solution environment is used with the appropriate working and counter electrodes. While certain electrode systems have a very low detection limit for iodine, they can suffer poor reproducibility due to absorption onto or possibly oxidation of the electrode surfaces. One electrode system has been found to offer good sensitivity and repeatability.

$H_2O_2$, a product of oxidase enzyme reactions, can be sensitively coupled to the reversible $I_2$-$I^-$ redox pair, offering a method for determining certain substrates of interest.

Sample volumes around 25 $\mu$l can be analysed at sampling rates typically of about 30 per hour, but as high as 80 per hour, by flow injection analysis.

REFERENCES

[1] Schwartz M. K., *Analytical Chemistry*, 1973, **45**, 739A.
[2] Betteridge D., *Analytical Chemistry*, 1978, **50**, 832A.
[3] Snyder L., Levin J., Stoy R. and Conetts A., *Analytical Chemistry*, 1976, **48**, 942A.
[4] Bergmeyer H.U. and Hagen A., *Analytical Chemistry*, 1972, **261**, 333.
[5] Ruzicka J. and Hansen E. H., *Analytica Chimica Acta*, 1976, 87, 353.
[6] Stewart K. K., Beecher G. R. and Hare P. E., *Analytical Biochemistry*, 1976, **70**, 167.
[7] Basson W. D. and Van Staden J. F., *Analyst*, 1978, **103**, 296.
[8] Nikolelis D. P. and Mottola H. A., *Analytical Chemistry*, 1978, **50**, 1665.
[9] Wolff C. H. and Mottola H. A., *Analytical Chemistry*, 1978, **50**, 95.
[10] Bailley P. L., *Analytical Chemistry*, 1978, **50**, 698A.
[11] Stulik K. and Vaclav V., *Journal of Electroanalytical Chemistry*, 1978, **70**, 253.
[12] Attiyat A. S. and Christian G. D., *Chemical, Biomedical and Environmental Instrumentation*, 1979, 9, 261.
[13] Attiyat A. S. and Christian G. D., *Analytica Chimica Acta*, 1979, **106**, 225.
[14] Attiyat, A. S., PhD Thesis, University of Washington, 1979.
[15] Pantel S. and Weisz H., *Analytica Chimica Acta*, 1977, **89**, 47.
[16] Pardue H. L., *Analytical Chemistry*, 1963, **35**, 1240.
[17] Gulberg E. L. and Christian G. D., *Chemical, Biomedical and Environmental Instrumentation*, 1979, 9, 277.
[18] Weetall H. H. in 'Methods in Enzymology' Ed. K. Mosbach, 1976, Vol. XLIV, Academic Press, London.
[19] Guilbault G. G. and Lubrano J. J., *Analytica Chimica Acta*, 1973, **64**, 439.
[20] Gulberg E. L. and Christian G. D., *Analytica Chimica Acta*, in press.
[21] Kissinger P. T., *Analytical Chemistry*, 1977, **49**, 488A.
[22] Lingane J. J., 'Electroanalytical Chemistry', 1958, Interscience Publishers, New York.

### Interfacing a titrator to a microcomputer for incremental or continuous modes of operation

**M. Danczíger and H. V. Malmstadt***
*School of Chemical Sciences, University of Illinois, Urbana, Illinois 61801, USA*

There are several microcomputer-controlled titrators now on the market. Some of these have been programmed to utilise many methods of endpoint detection, including the incremental titrant delivery and calculation techniques. However, these incremental methods are not readily implemented on older titrators. It is the purpose in this paper to describe some rather simple interface designs and automation methodology that enable a few conventional titration modules to be interconnected with a microcomputer so as to provide an 'intelligent' and versatile automated titrator. This system is then used to provide some comparisons of the various incremental titrant delivery and calculation modes. It can also be used in the continuous delivery mode to a preset [1] or derivative [2,3] endpoint. Several concepts of a microcomputer-controlled titrator and selection of an endpoint calculation technique are illustrated.

Although the theoretical aspects of the Kolthoff [4], Fortuin [5], Wolf [6], Keller-Richter [7], and Bartscher [8] incremental methods have been discussed, there is little information in the literature on practical comparisons. The automated titrator described here has enabled hundreds of unbiased titration results to be printed out rapidly for the various incremental techniques. Results are presented and discussed for a weak acid-strong base and a precipitation titration. The incremental methods are compared on the basis of the experimental results obtained.

*Correspondence to this author

### Instrumentation

A block diagram of the automated titrator is shown in Figure 1. An ADD-8080 microcomputer [9] provides the "intelligence" for the titrator. It is based on the 8080A microprocessor (Intel Corp., Santa Clara, Calif. 95051, USA). The microcomputer has 10K bytes of programmable read only memory (PROM) which contain a BASIC interpreter (a modification of BASIC/5, Processor Technology Corp., Emeryville, Calif. 94608, USA), a monitor program to facilitate machine language programming, and several utility programs. Also present are 15K bytes of read-write memory (RWM) which are used to store BASIC user programs in machine language and data. An arithmetic processing unit (AM9511 APU, Advanced Micro Devices, Inc., Sunnyvale, Calif. 94086, USA),is available to perform calculations that would be too time-consuming or cumbersome if done on the microprocessor. A conventional teletypewriter provides interaction with the operator.

The following section presents in detail the interface developed specifically for the automated titrator. Firstly the modification of the constant-rate burette including volume encoding and the software program that provide precise increments of titrant are described. Secondly, details of the voltage-to-frequency (V-to-F) converter and counter interface between the electrodes and the microcomputer are given. A chart recorder interface enabling hard-copy output of titration curves is also described.

### Burette modification

The burette (No. S-11120, Model C Constant Rate Burette, Sargent-Welch Scientific Co., Skokie, Illinois 60076, USA) is normally operated at constant speed. It has two motors to drive the plunger. One is a synchronous motor for constant rate delivery. The other is a bidirectional rapid drive motor which is used for emptying, rinsing, flushing, and filling the burette. This motor would not normally be used to deliver titrant to the reaction vessel.

Details of the modification of the burette and its computer interface are shown in Figure 2. Computer control of the burette requires simulated closing of the front panel switches using relays. Switch S3 selects either manual or computer control. When this is in the computer position, power is directed to the delivery motor, or the rapid drive motor, or neither motor, depending on the states of relays K1 and K2. A reliable interface must be devised to allow the computer to drive these relays as well as the solenoid valve, K3. This switches the burette to either the delivery tip or the titrant reservoir. It is particularly important that the inductive spike that results when a relay coil is released does not interfere with the computer. To prevent this, separate power supplies are provided to power the relays and solenoid valve. These power supplies are based on three-pin voltage regulators (Nos. 7805 and 7812, Fairchild Camera and Instrument Corp., Mountain View, California 94042, USA) which can provide up to one ampere at their regulated voltage. The relays and solenoid valve each require about 200 mA. Communication of control signals from the computer is achieved through optically-coupled isolators (4N26). A computer bit is output through a parallel port and this is fed to transistor Q1. This transistor can operate over the few feet of cable between the computer and the burette. When the computer bit is HI, transistor Q1 is on, which turns the diode of the optical isolator off. This turns off the phototransistor of the optical isolator which turns on transistor Q4, and the relay coil is energised. Conversely, when the computer bit is LO, the coil is not energised. The other two circuits operate in a similar fashion.

### Volume encoder

A wheel with alternate opaque and transparent radial sectors was mounted on the drive shaft of the burette to provide an accurate measure of the volume delivered. This wheel was fabricated by gluing a photographic negative of 20 black and white sectors onto a plexiglass disc (2.5 in diameter, 1/16 in thick). The wheel and a photon coupled interrupter module (No. H13B1, General Electric, Syracuse, New York 13201, USA) produce twenty light pulses per revolution of the drive shaft. There are 100 revolutions of the shaft per millilitre delivered.

The interface of the volume encoder to the computer is shown in Figure 3. Schmitt trigger gates configured as an exclusive-or monostable [10,11], produce a short output pulse (100 $\mu$ sec) for each dark-to-light or light-to-dark transition at the interrupter module. This effectively acts as a frequency doubler. Thus 4000 electronic pulses are produced for each millilitre of titrant delivered. These pulses are accumulated in a 16-bit down counter. This is one of three such counters available in a single integrated circuit package (8253 Programmable Interval Timer (PIT), Intel Corp.), which is physically located at the microcomputer. To ensure high noise immunity for the volume encoder signal, a line driver and receiver are used in the differential mode, and are connected by a twisted pair cable.

### Operation in the incremental mode

In normal constant-speed operation the burette will deliver 0.1 ml in 6 seconds with a 10 ml capacity cylinder. Under

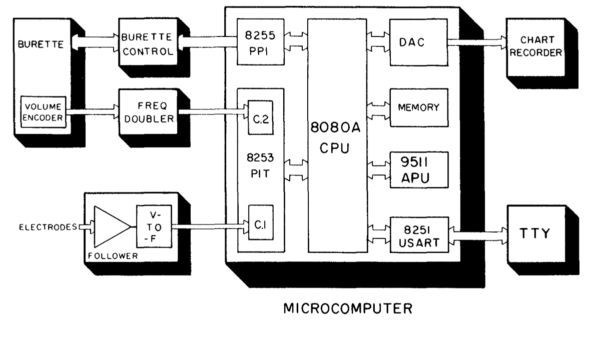

*Figure 1. Block diagram of automated titrator.*

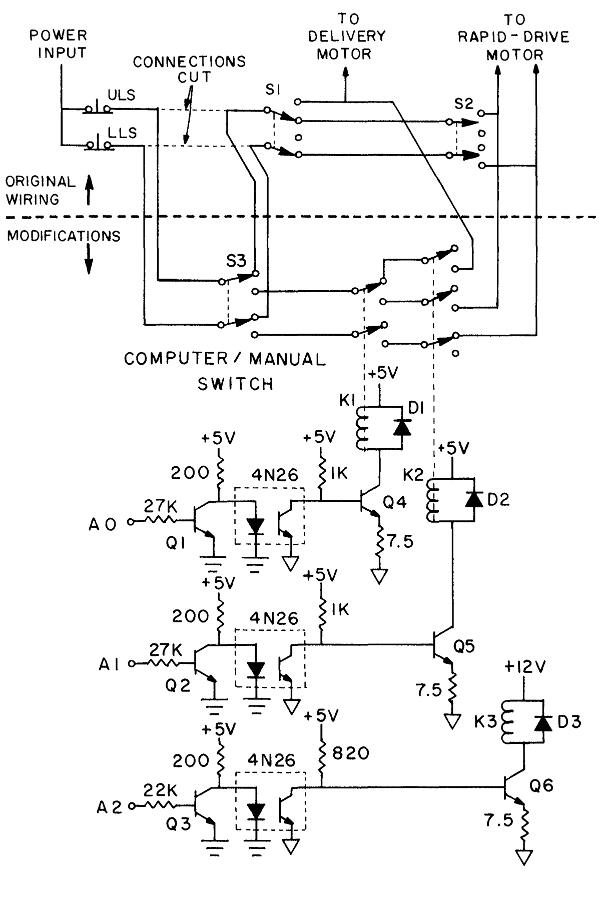

*Figure 2. Schematic diagram of burette interface. Key to components: ULS, upper limit switch; LLS, lower limit switch; S1, delivery switch; S2, rapid drive switch; Q1, Q2, Q3, MPS 6515; Q4, Q5, Q6, MJE 521; D1, D2, D3, IN3193; K1, K2, Sigma relays 67 REV4-5DC; K3, solenoid valve 1-17-900 (General Valve Corp., E. Hanover, New Jersey, USA); A0, A1, A2, bits output form the computer through a parallel port; all resistances are in ohms, 1/4 watt, 5%.*

computer control, the burette can deliver an accurate 0.1 ml increment of titrant about five times faster. The rapid drive motor is used for the major portion of the increment.

To deliver one increment of titrant, the following sequence takes place: 1) The rapid drive motor is turned on for a short time (220 msec for a 0.1 ml increment) to deliver most of the increment. 2) The rapid drive motor is turned on in the reverse direction for 80 msec. This quickly brakes the motor without causing the plunger to start moving backwards. 3) The delivery motor is turned on to complete delivery of the increment. The rapid drive motor cannot be used for the entire increment because it cannot be stopped as quickly or as reproducibly as the delivery motor when approaching the terminal count (400 for 0.1 ml).

Using this method, the burette can deliver each 0.1 ml increment in 1.3 sec. A 0.2 ml increment can be delivered in about 1.5 sec because a larger proportion of it is delivered with the rapid drive motor.

**Electrode interface**

The interface to input electrode potentials to the computer, Figure 4a, uses an operational amplifier (CA3140, RCA Corp., Somerville, New Jersey 08876, USA) with a very high input impedance (1.5 TΩ) so that the measurement circuit does not load the electrode. The amplifier is set up as a voltage follower with variable gain. The output of the follower ($V_{out}$) is directly accessible at the electrode interface. This allows chart recordings of titration curves to be made directly without computer involvement. When the switch in the gain circuit of the follower is opened, the follower has a gain of one. An inexpensive V-to-F converter used in conjunction with the two remaining 16-bit counters in the 8253 PIT performs the analogue-to-digital conversion. Extremely high data rates are not encountered in the course of collecting volume and voltage values during a titration. For this reason, a more expensive type of analogue-to-digital converter need not be used. The V-to-F converter (A-8400, Intech Function Modules, Santa Clara, California 95050, USA) is configured to accept both positive and negative potentials by adding an offset voltage to its summing junction [12].

Each of the three 16-bit counters in the 8253 PIT has two inputs, called gate and clock, and one output, which can be directly accessed by the user. Total counts can be preloaded or read by the 8080A CPU through its data bus. This versatile integrated circuit can have each of the three counters independently programmed into any one of six modes of operation [13].

Counters 0 and 1 are easily interconnected to be a frequency measuring system. Counter 0 is programmed as a one-shot device (Mode 1). It is preloaded with n counts by the software. It starts counting down on the first falling edge at its clock input after the rising edge at its gate input (see Figure 4b). The output of counter 0 will then remain L0 until n counts have elapsed. For example, if the preload value is 50,000 and the frequency at the clock input is 1,000 MHz, then an output pulse of 50.00 msec is produced. The output of counter 0, after inversion, becomes the gate control for counter 1. Counter 1 is preloaded with a full count and will not reach a count of 0 before its gate is closed. In this manner, the counts from the V-to-F converter are counted over very precise time intervals that depend only on the stability of the oscillator fed to clock 0. The output of counter 0 is also input to the CPU through one bit of a parallel port. The rising edge here indicates that one measurement cycle has ended.

**Chart recorder interface**

When titrations are run in the continuous mode, a larger number of points are stored in memory. These can include the first (and higher) derivatives of titration curves as well as the curves themselves. It is often very helpful to inspect the titration curve and its derivative(s) that are stored in memory. This is particularly true when experimental parameters are being modified. Figure 5 shows the chart recorder interface that is used to provide this capability. A 10-bit multiplying digital-to-analogue converter, (DAC 331-10, Hybrid Systems Corp., Bedford, Massachusetts 01730, USA) is used with a 10-volt precision voltage reference source (REF-01, Precision Monolithics Inc., Santa Clara, California 95050, USA). This produces a full scale voltage range of 0 to −10V at the output of A1. A2 is an inverting amplifier with a gain of −0.0100 so that the signal at the output of A2 has a full scale range of 0 to +100 mv. Most chart recorders accept an input having this range. The ratio of the feedback resistor to the input resistor of A2 can be changed to provide other output ranges.

An assembly language program is used to output a titration curve from memory. For each point, the eight most significant bits are sent out to Port B of the 8255 PPI,

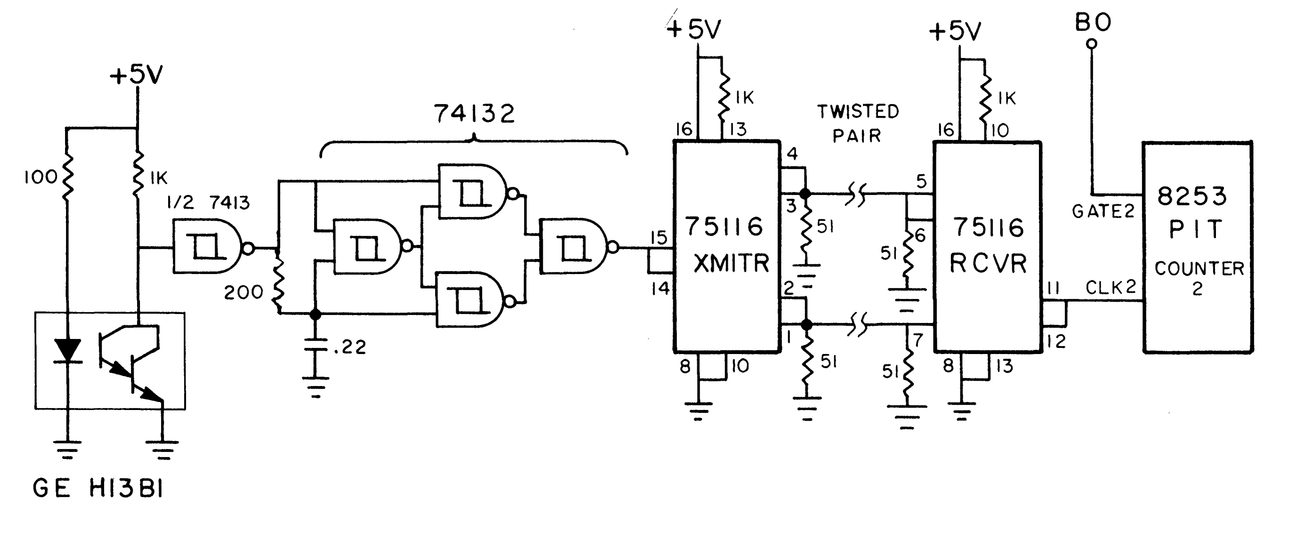

*Figure 3. Schematic diagram of volume encoder interface. Key to components: H13B1, photon coupled interrupter module; 75116 XMITR, 75116 RCVR, differential line transceivers used as transmitter and receiver, respectively; B0, output bit from 8255 port; all resistances are in ohms, 1/4 watt, 5%; capacitance is in microfarads.*

followed immediately by the two least significant bits being sent to Port C. There is then a delay before the next 10-bit point is sent out. The length of this delay is adjusted to suit the number of points that are to be output. Typically, an 8 msec delay is used and this allows 4096 points to be sent out in about 33 sec. This would be the case for data accumulated with the burette running at constant speed and data points being collected continuously. This results in a smooth titration curve. For titrations in the incremental mode, there are inherently only a few data points in the endpoint region, and they would not normally be plotted.

**Table 1. Calibration of electrode interface**

| $V_{in}$, V | $F_{out}$, KHZ* | % RSD |
|---|---|---|
| +0.500 | 113.87 | 0.026 |
| +0.400 | 98.48 | 0.069 |
| +0.300 | 83.07 | 0.091 |
| +0.2000 | 67.80 | 0.067 |
| +0.1000 | 52.28 | 0.045 |
| 0.000 | 36.94 | 0.019 |
| −0.1001 | 21.45 | 0.061 |
| −0.2000 | 6.06 | 0.090 |

Linear regression: slope = 154.02 KHz/v
intercept = 36.89 KHz
correlation coefficient = 0.999999

**Each output frequency is the mean of five measurements.*

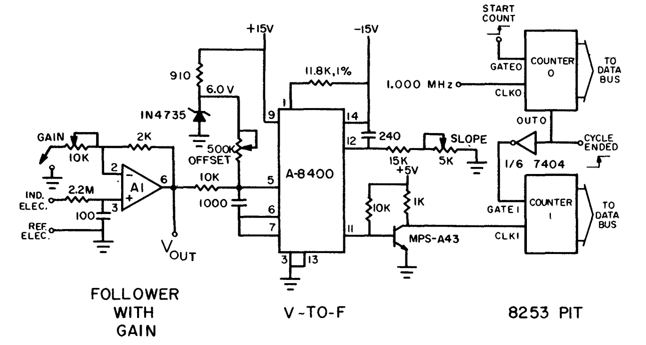

*Figure 4a. Electrode interface schematic diagram. Key to components: A1, CA3140; all resistances are in ohms, 1/4 watt, 5% unless otherwise specified; all capacitances are in picofarads.*

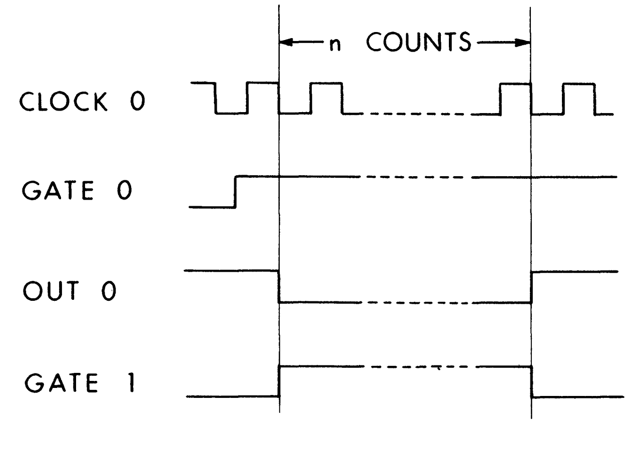

*Figure 4b. Timing diagram for 8253 PIT counters 0 and 1.*

### System evaluation

The linearity and precision of both the electrode interface and the incremental method of delivery were tested and the results presented. Table 1 shows the output frequency for the electrode interface as a function of input potential. The data show that excellent linearity and precision can be attained. Different voltage ranges can be obtained by adjusting the gain of the voltage follower or the slope or offset of the V-to-F converter.

To test that the incremental mode of delivery does deliver precise and accurate amounts of titrant, deliveries of water were made into a weighing bottle which was accurately weighed before and after delivery. The uncertainty in a 0.1 ml increment was found to be 0.3 $\mu$l. Table 2 shows results for larger volumes.

### Titration conditions

Analytical reagent grade chemicals were used for the preparation of all reagents. Acetic acid (Allied Chemical, Specialty Chemicals Division, Morristown, New Jersey, USA) and sodium hydroxide (Mallinckrodt, Inc., St. Louis, Missouri 63147, USA) were prepared using freshly boiled distilled water. Sodium chloride (Fisher Scientific Co., Fair Lawn, New Jersey 07410, USA) and silver nitrate volumetric concentrate (Acculute, Anachemia Chemicals Ltd., Montreal, Canada) were dissolved in distilled water. The silver nitrate solution was stored in the dark to minimise the possibility of photodecomposition. A combination pH electrode (No. 13-639-92, Fisher Scientific Company, Pittsburgh, Pennsylvania 15219, USA) was used for the acid-base titrations. For the precipitation titrations, a Ag/AgCl electrode (No. S-29718, Sargent-Welch Scientific Co.) was referred to a

**Table 2. Linearity of incremental delivery (0.1 ml increments)**

| Volume, ml | Mass, gm* | % RSD |
|---|---|---|
| 0.5 | 0.4925 | .22 |
| 1.0 | 0.9856 | .13 |
| 2.0 | 1.9670 | .12 |
| 3.0 | 2.9498 | .09 |
| 4.0 | 3.9404 | .11 |
| 5.0 | 4.9289 | .15 |
| 6.0 | 5.9093 | .03 |

Linear regression: slope = 0.9853 gm/ml
intercept = -0.0016 gm
correlation coefficient = 0.999997

**Each reported mass is the mean of six weighings.*

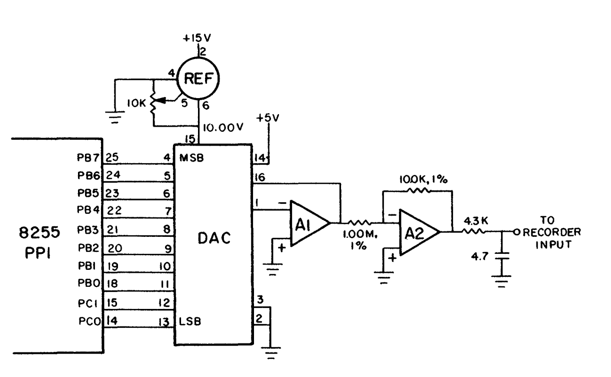

*Figure 5. Chart recorder interface schematic diagram. Key to components: REF, REF-01 10 volt precision voltage reference source; DAC, 331-10 digital to analogue converter. A1, A2, CA3140; all resistances are in ohms, 1/4 watt, 5% unless otherwise specified; capacitance is in microfarads.*

saturated calomel electrode (No. 30490, Sargent-Welch Scientific Co.). A salt bridge (2M $KNO_3$ in 4% agar) was used to prevent precipitation of silver by chloride ions from the calomel electrode.

Incremental titration results were obtained for five calculation methods for the acid-base titrations and for four methods for the precipitation titrations. In incremental titrations, an increment of titrant is added as described. Then the voltage across the electrode pair is monitored either for a fixed time or until it 'stablises'. To decide when stability has been reached, the computer acquires a voltage measurement (by integrating the count from the V-to-F converter over 50 msec), delays one-half second, then takes a second point. If the two voltages differ by less than a preset amount, the computer decides that stability has been reached. If not, the computer continues testing in this way until the difference falls below the preset amount. This was the mode of operation for the silver chloride precipitation titrations. The criterion for stability in this case was a rate of change of potential of <1.5 mV/sec. When the voltage has stabilised within this limit, the volume/voltage values for that increment are stored in memory, and the next increment is added. When five increments have been added beyond the one with the largest potential jump (Δo), the titration is stopped. Endpoints are then calculated using the methods of Fortuin [5], Wolf [6], Keller-Richter [7], and Bartscher [8]. Results for these titrations are presented in Table 3.

Another approach to incremental titrations that can be used involves waiting a fixed time after the addition of each increment rather than waiting for the rate of change of electrode potential to fall below a certain level. This is the mode of operation for the acetic acid-sodium hydroxide titrations. The titrations are started by adding 0.10ml increments of titrant. Immediately following the addition of each increment, the potential difference is measured and stored in memory. Then a new cycle of titrant addition and voltage measurement is begun. When two successive voltages differ by at least a certain amount (10 mV in this case) the computer recognises that it is in the equivalence point region of the titration. The titrant increment is then automatically reduced to 0.05ml. Each increment is followed by a delay of 3.0 sec, after which the potential difference corresponding to that increment is measured. This combination of larger, more rapid increments for most of the titration, and smaller, slower increments only in the equivalence point region provides rapid titrations as well as an accuracy determination of the endpoint volume.

**Table 3. Comparison of incremental methods for titration of sodium chloride with silver nitrate.**

| 0.035 N NaCl volume, ml | Method | 0.1 N $AgNO_3$ titrant volume, ml* | Precision % RSD |
|---|---|---|---|
| 5.00 | Fortuin (F) | 1.739 | 0.11 |
| | Wolf (W) | 1.740 | 0.11 |
| | Keller-Richter(KR) | 1.740 | 0.10 |
| | Bartscher (B) | 1.742 | 0.20 |
| 10.00 | F | 3.479 | 0.10 |
| | W | 3.478 | 0.10 |
| | KR | 3.479 | 0.10 |
| | B | 3.478 | 0.17 |
| 15.00 | F | 5.214 | 0.13 |
| | W | 5.215 | 0.14 |
| | KR | 5.214 | 0.16 |
| | B | 5.217 | 0.18 |
| 20.00 | F | 6.964 | 0.11 |
| | W | 6.962 | 0.09 |
| | KR | 6.964 | 0.12 |
| | B | 6.964 | 0.11 |

**Mean value for eight titrations; 0.10 ml increments delivered throughout the titration.*

Specific experimental parameters must be chosen to suit the titration at hand. For example, how does one select the increment size that should be used? As the increments are made progressively larger, the titration is performed more quickly. However, the ability of the calculation methods to accurately determine the endpoint volume generally improves as the increments are made smaller. Because the appropriate increment size depends on the specific titration reaction and the titrator setup, it is best to determine the optimum increment size experimentally.

If a fixed time is used between the addition of an increment and the measurement of the 'equilibrium' potential, what is the proper length of time to wait? If the electrode is slow to come to its equilibrium potential, or if the chemical reaction itself is slow, it is possible to measure potential differences too soon. This might also be the case if the mixing is inefficient. However, if the delay before the voltage measurement becomes too long, it is possible for the electrode potential to drift, particularly if the indicator electrode does not show a well-defined response to the chemical reaction being monitored. For small volumes of titrant and long delay times, the diffusion of titrant from the delivery tip could also cause errors. In this respect, the fixed delay time method is less susceptible to errors than the method of waiting for a certain rate of change of electrode potential. These are some of the considerations that should be noted when incremental titrations are to be used.

Continuous titrations can also be easily performed with the automated titrator. Titrant is delivered at a constant speed and potential differences of the electrode pair are measured 'continuously' for 50 msec integration periods of the V-to-F converter, and the computer stores the 50 msec points in memory during the course of the titration. At the conclusion of the data collection, the endpoint can be calculated as the maximum in the first derivative of the curve or the zero-crossing of the second derivative by applying Savitzky-Golay convolutes [14]. Alternatively, the

**Table 4. Comparison of incremental methods for titration of acetic acid with sodium hydroxide.**

| 0.025 N $CH_3COOH$ volume, ml | Method | 0.1 N NaOH titrant volume, ml* | Precision % RSD |
|---|---|---|---|
| 5.00 | Kolthoff (K) | 1.274 | 0.10 |
| | Fortuin (F) | 1.263 | 0.29 |
| | Wolf (W) | 1.263 | 0.29 |
| | Keller-Richter(KR) | 1.262 | 0.36 |
| | Bartscher (B) | 1.262 | 0.39 |
| 10.00 | K | 2.527 | 0.09 |
| | F | 2.530 | 0.15 |
| | W | 2.530 | 0.15 |
| | KR | 2.530 | 0.15 |
| | B | 1.535 | 0.11 |
| 15.00 | K | 3.784 | 0.03 |
| | F | 3.797 | 0.03 |
| | W | 3.797 | 0.04 |
| | KR | 3.798 | 0.04 |
| | B | 3.797 | 0.03 |
| 20.00 | K | 5.045 | 0.29 |
| | F | 5.052 | 0.09 |
| | W | 5.052 | 0.10 |
| | KR | 5.053 | 0.08 |
| | B | 5.054 | 0.04 |
| 25.00 | K | 6.328 | 0.02 |
| | F | 6.320 | 0.08 |
| | W | 6.320 | 0.08 |
| | KR | 6.320 | 0.09 |
| | B | 6.319 | 0.09 |

**Mean value for five titrations; 0.10 ml increments delivered initially, followed by automatic switching to 0.05 ml increments in the region of the equivalence point.*

computer can implement a digital version of endpoint detection schemes performed in the past by analogue circuitry. For example, a 'dead-stop' titration [1] can be performed by comparing the incoming voltages to the preset endpoint value stored in memory.

The high-speed computation capability of the computer also allows it to calculate first or second derivatives 'on the fly' and end the titration when it recognises that the endpoint has been passed. These approaches usually take somewhat less time than the storage of the entire continuous titration curve and the calculation of the endpoint 'after the fact'. However, all continuous addition methods can give large blanks and if the chemical reaction is slow, the stirring is inefficient, or the electrodes respond slowly erroneous results can occur. In such case the incremental methods are generally advantageous and provide more accurate results more rapidly. The automated microprocessor-controlled titrators make these incremental methods very practical.

### Discussion of Results

The results for the automated precipitation titration of chloride with silver nitrate by four of the incremental fixed-volume endpoint methods are shown in Table 3. The results in Table 4 are for the titration of acetic acid with sodium hydroxide using five different incremental methods for calculation of the endpoint. The Fortuin [5], Wolf [6], and Keller-Richter [7] algorithms are all based on the four data points encompassing the three largest potential jumps in the equivalence point region. The similarity of the calculation formulas is reflected in the results printed out for these methods. For any individual titration, the Fortuin and Wolf results are virtually identical. The Keller-Richter result is most often within 0.1% of the Fortuin and Wolf methods. However, the more recently proposed method of Bartscher [8] is fundamentally different from the other three methods. It uses the six points surrounding the largest potential jump rather than just the inner four, and factors obtained from the asymmetry of the curve. The mean values shown for the Bartscher method in Tables 3 and 4 in all cases agree closely with the other three methods. In some instances, the Bartscher method showed slightly poorer precision than the Fortuin, Wolf, and Keller-Richter methods, but most often agreed within 0.1%. For the titrations in Table 4, the results were also calculated for the Kolthoff method [4]. This is simply a linear interpolation which calculates the inflection point of the curve based on the zero-crossing of the second difference. Except for the value at the 20.00 ml aliquot (which was due to one outlying result) the precision for the Kolthoff method was 0.10% or better. This was true even at the 5.00 ml aliquot where the other methods had standard deviations three times larger.

As long as a titration is not subject to determinate error, a blank should not be observed. This is borne out for the data in Tables 3 and 4. Linear regressions performed on the results for the different methods show intercepts which range from -0.003 to +0.004 ml. This is close to the limits of the precision of the overall measurements. The slopes calculated by the linear regressions (which are representative of the titer) are identical for all the methods in each table. Correlation coefficients range from 0.999984 to 0.999999, showing the excellent linearity obtained.

Although the incremental acid-base and precipitation titrations were performed in two essentially different manners, the speeds for both were approximately the same. The 5.00 ml aliquots of sodium chloride required about 65 seconds to titrate to an endpoint of 1.7 ml. The most concentrated aliquots, which required 7.0 ml, took 4 minutes to titrate. The acetic acid titrations, requiring from 1.3 to 6.3 ml of titrant, took from 70 seconds to 3.3 minutes.

The designs presented here illustrate how a microcomputer-controlled titrator can be developed from basic modules and simple interfacing circuits. The precise and accurate data demonstrate that this versatile titrator is ideally suited for efficient characterisation of endpoint detection methods as well as for rapid routine titrations.

ACKNOWLEDGEMENT

This project was supported in part by research grant NIH HEW PHS GM21984-05.

REFERENCES

[1] Lingane, J. J., "Electroanalytical Chemistry, Second Edition," Interscience Publishers, Inc, New York, 1957, 158.

[2] Malmstadt, H. V. and Fett, E. R., *Analytical Chemistry*, 1954, **26**, 1348.

[3] Malmstadt, H. V. and Fett, E. R., *Analytical Chemistry*, 1955, **27**, 1757.

[4] Kolthoff, I. M. and Furmann, N. H., "Potentiometric Titrations", Wiley, New York, 1949.

[5] Fortuin, J. M. H., *Analytica Chimica Acta*, 1961, **24**, 175.

[6] Wolf, S., *Fresenius' Zeitschrift fur Analytische Chemie*, 1970, **250**, 13.

[7] Keller, H., and Richter, W., *Metrohm Bulletin*, 1971, **2**, 173.

[8] Bartscher, W., *Fresenius' Zeitschrift fur Analytische Chemie*, 1979, **297**, 132.

[9] Avery, J. and Lovse, D., "The ADD-8080 Microprocessor Manual," Department of Chemistry, University of Illinois, Urbana, Illinois 1977.

[10] Alfke, P., *Fairchild Journal of Semiconductor Progress*, July/August 1977, 26.

[11] Malmstadt, H. V., Enke, C. G., and Crouch, S. R. "Digital and Analogue Data Conversions, Module 3," W. A. Benjamin, Inc., Menlo Park, California, 1978, 244.

[12] "A-8400 User Guide," Intech Function Modules, Santa Clara, California 95050, USA.

[13] "8080 Microcomputer Peripheral User's Manual," Intel Corp., 3065 Bowers Avenue, Santa Clara, California 95051, 14.

[14] Savitzky, A. and Golay, M. J. E., *Analytical Chemistry*, 1964, **36**, 1627.

